# The Education of Mothers of Families; or the Civilization of the Human Race by Women

**Published:** 1842-07-01

**Authors:** 


THE EDUCATION OF MOTHERS OF FAMILIES; OR THE CIVILIZATION OF THE HUMAN RACE BY WOMEN. Translated from the French of *Aimé-Martin*, M.D. by *Ed. Lee*, Esq. 8vo. pp. 384. Whittaker and Co. 1842.

THIS is a very strange production; and although medical men are much concerned in the subject of *physical* education, both in males and females —yet in respect to *moral* instruction, the clergyman is the person who has most time and the best means of superintending that. Dr. Martin's sentiments are greatly too sublime and unintelligible for us—and, we suspect, for the generality of readers in this country. The following passage contains a whole chapter, and is by no means the worst, nor the shortest of the 34 chapters of which the work consists.

SENSE OF INFINITY—A FACULTY OF THE SOUL.

"Everything is transitory upon the earth; all speaks to us of our nothingness: *life is composed of days which are no more, and the present is but the future which passes by.* Still, if time were only to spare our reminiscences; but after the transports of joy and the pains of grief, indifference and oblivion supervene. Our existence is effaced even in our memory; we depart by piecemeal, and these portions detaching themselves day by day, disappear in proportion as we advance. Thus the past dies, the present vanishes, and the future is but a hope. A hope —O mortal, this is thy greatness! Amidst this world of destruction, in the presence of death and oblivion, when all around thee perishes, thou hopest a life which will never end! The word eternity does not astonish thy soul: it responds to it by that of infinity, the sublime sentiment which detaches us from time and space, to transport us to the bosom of God.

It is because the sense of infinity exists in us, that nothing which is finite can satisfy our souls.

The horror of annihilation is a revelation of infinity.

But what is infinity? all my efforts to conceive it are useless. It is equally impossible for me to deny it or to understand it. What I know is, that out of infinity there is nothing, or, to express myself better, that all is in infinity. Guided by this feeble light, I lay down a number, to which I add other numbers; I fill immensity with my calculations.—Useless trouble! The sum constantly increasing, is only composed of finite things: I must always refer to its two extremes, the beginning and the end: but there, casting my eyes on this side and on that, I perceive no end, no beginning; that which the figures of arithmetic pursue without ever attaining, that which is before and after, that which is everywhere and always, constitutes infinity.

The sentiment of infinity gives us the idea of all that which cannot be attained by the senses: it realises for us the unknown.

God is infinity: it is God which thou seekest, O my soul, since nothing of that which is finite can satisfy thee here—below. Thou detachest thyself from all the joys of earth, because all these joys have an end. Thou placest thy dependence solely upon this infinity, which is beyond all our passions, and which is at the same time thy hope, thy light, and thy satisfaction.

Thus, man is the point of union between nature and its Creator: all that which he experiences beyond his earthly desires is an announcement of eternity. It is by means of intelligence and love that nature arrives at him: it is by the sense of the beautiful and the infinite, that he arrives at God. The chain commenced on earth does not break, but ascends to lose itself in heaven.

The sense of infinity—a faculty of the soul. The third light which radiates towards God." 110.

Nine tenths of the whole volume are composed of such brilliant meteors as the foregoing chapter. There are only two or three short chapters which actually bear on the subject of female education. The following passage in chapter 6, gives us the substance of female education as it at present exists.

"Since the periods of Fénelon and Rousseau there has been progress among men, and the education of women has consequently improved. We now no longer discuss the question, whether they should be instructed, or the amount of the instruction which should be allowed them. We consent to develope their intelligence. We go further, and give them the talents of artists, and of doctors of sciences; they skim, if we may so speak, encyclopædial studies, but in these studies there is nothing which leads them to think with their own thoughts. When, therefore, the passions arrive, to which it is not too much to oppose habits of virtue, the powers of the soul, and the principles of religion, they find hands skilful upon the piano, a memory which recites, and a soul which sleeps. Such is, with a few rare exceptions, the woman which the age gives us, with her petty devotional practices, her school morality, her mechanical talents, her love of pleasure, the ignorance of the affairs of life, and the want of loving and of being beloved." 31.

But what is the object of the education of women? To get married. This is the summum bonum for which the mother plots and the daughter studies. Both of them well know that this paramount object is not so readily obtained by mathematics as by music—by natural philosophy as by drawing—by walking in the day as by waltzing at night. These three studies, especially MUSIC, engross four-fifths of a young lady's time, and both solid instruction and health are sacrificed. The splendid instances of exception to this rule are as dust in the balance. Do we blame mothers and daughters for this kind of education? Not at all. People who manufacture wares for sale consult the taste of their customers rather than their own. If men will have the harp and piano, with broken health, they must also have the physician and apothecary as daily visitors to the end of the chapter.

This is the education of the higher classes of society. That of the middle classes consists in eager and sometimes successful imitation of what their superiors enjoy. As to the lower orders, they may be said to have no education at all.*

"Thus, in proportion as we seek the end, everything disappears; nothing for

---

* See the Report of the Educational Committee, or Ed. Review for April.

private happiness, nothing for the general prosperity. The world remains, and it is to that point, in fact, towards which all our previsions are directed. The object is more to please the world than to resist it; to shine, to reign. Vanity; such is the object which the tenderest mothers do not cease to show their daughters, and upon which rock the world, which cheers them on, sees them wreck themselves with indifference. Vanity in dress, vanity in agreeable talents, vanity in instruction. Be handsome, be polite, people see you; be gentle, be submissive, people hear you, says a mother to her daughter; that is to say, let appearance always take the place of reality. The soul, like the body, has its light dresses, to which we are accustomed from the cradle. The evil is not cured, it is concealed; the character is not changed, it is disguised; thus vanity covers all: to *seem* not to *be*, constitutes the aim of education." 34.

It is the 10th chapter of the work, consisting of eleven pages, that contains "The Education of Mothers." The others treat de omnibus rebus et multis aliis. Thus for instance we have a chapter on each of the following subjects:—"Know thyself"—"Of Instinct"—"Philosophical Physiology"—"Sense of Infinity"—"Immortality of the Soul"—"The Soul of Nations"—"The Unity of God"—"Love"—"Re-action equal to Action"—"Life and Death"—"Death not a Punishment"—"Hopes of Futurity," &c. &c.

But to return to the chapter in question. Our author having censured the existing education of the young female, without proposing any specific reforms, takes her into his own keeping when she has just left her parents, and become a wife. She now, he says, begins to inquire how she may best educate her offspring. The ladies of France must be very different from those of England, if this idea or solicitude is the first to engross their minds after the nuptial ceremony. But let that pass.

"The first thought which she should be led to entertain, is to occupy herself a little less about that which she ought to teach, and a little more about that with which she ought to inspire him. Many other persons may render him learned, she alone can render him virtuous. Let the mother take charge of the soul, in order to be able, at a future day, to direct the intellect!" 48.

"This," says he, "is the summary of the education of mothers of families."

"Their mission consists in introducing our childhood into this world as into a holy temple, where the soul looks into itself, and knows itself to be in the presence of its God." 49.

In the next page we have this sublime, and to us rather novel effusion.

"Is it not philosophy which unites man to man, and the human race to God? These questions, so vast, of annihilation and eternity, which absorb the meditations of the philosopher, how often have I not found them occupying the villager in his cot, and the soldier in his bivouac! I know no metaphysics more transcendental than those which are formed in a camp, on the eve of a great battle. *What silent contemplations of infinite worlds—what thoughts directed towards invisible creations—what ardent prayers towards that celestial life which was forgotten yesterday, but which is now something more than a hope!* If a ball strikes me to-morrow, all these luminaries will shine below me! *God reveals himself to those about to die;* and from amidst this crowd, which no religion humanizes, no instruction softens; from this impure sink of debauchery, of crimes, and of impiety, arises all at once an immortal thought, which penetrates to the depths of the soul, and transports it to the bosom of God." 50.

The following is the best passage in this chapter, or, in the whole of the author's portion of the book.

"Thus we shall arrive at the most important part of this book—the moral study of the Gospel. I say the most important, for all education, which has not religion for its basis, renders man incomplete, and succeeds at best but in forming an intelligent animal. It is a mistake to suppose that man is great by means of science; he is only great—he is only man, by the knowledge of God. Beyond this we only see his circumscribed life, and a philosophy without light.

Wherefore does such universal egotism exist? Whence arises the love of gold? the love of power? the love of vengeance, instead of the love of humanity? Whence arises so much ambition, which engenders so many crimes? Whence so many murders and adulteries? and so much ingratitude, calumny, and depravation? From two causes, error and misery; and there is only one remedy—religion." 56.

Such a sentiment from the lips or the pen of a Frenchman, is a most rare phenomenon!

About sixty pages of this volume are dedicated to the subject of "the Prevailing Methods of Education," by Mr. Lee himself, in the form chiefly of a compilation from the scattered writings and opinions of various eminent personages—principally English.

"But as there exists much diversity of opinion upon the subject, I have preferred citing the observations of some authorities than too freely obtruding my own, (which are chiefly restricted to considering the matter in a hygienic point of view) and have consequently quoted pretty largely from publications in which education is treated of in its general and medical bearings, considering that these quotations would have much greater weight than the opinions of an individual, (especially of so humble a one as myself,) and that from the aggregate, each reader would be better able to form a correct judgment, as well as to perceive more clearly the source whence arise some of the evils which afflict the community, and the most likely means of diminishing the frequency of their occurrence." 328.

This is the really valuable part of the volume, and Mr. Lee's remarks which connect the various links of this chain of quotations, are always judicious, and often highly important. We only regret that Mr. Lee did not take a more ample part in the construction of the volume; for, although it might not, in that case, have contained so many brilliant passages, emulating those of Rousseau, or rather St. Pierre; yet it would have presented a denser mass of solid English sense and practical observation.

